# Ammonia Affects Astroglial Proliferation in Culture

**DOI:** 10.1371/journal.pone.0139619

**Published:** 2015-09-30

**Authors:** Guillermo Bodega, Berta Segura, Sergio Ciordia, María del Carmen Mena, Luis Andrés López-Fernández, María Isabel García, Isabel Trabado, Isabel Suárez

**Affiliations:** 1 Departamento de Biomedicina y Biotecnología, Universidad de Alcalá, 28871 Alcalá de Henares, Madrid, Spain; 2 Instituto de Salud Carlos III, UFIEC, Unidad de Neuro-Oncología, 28220 Majadahonda, Madrid, Spain; 3 Proteomics, Centro Nacional de Biotecnología/CSIC, Campus de Cantoblanco, Madrid, Spain; 4 Servicio de Farmacia, Instituto de Investigación Sanitaria Gregorio Marañón, Hospital General Universitario Gregorio Marañón, Madrid, Spain; 5 Unidad de Cultivos, Facultad de Medicina, Universidad de Alcalá, 28871 Alcalá de Henares, Madrid, Spain; University of Alabama at Birmingham, UNITED STATES

## Abstract

Primary cultures of rat astroglial cells were exposed to 1, 3 and 5 mM NH_4_Cl for up to 10 days. Dose- and time-dependent reductions in cell numbers were seen, plus an increase in the proportion of cells in the S phase. The DNA content was reduced in the treated cells, and BrdU incorporation diminished. However, neither ammonia nor ammonia plus glutamine had any effect on DNA polymerase activity. iTRAQ analysis showed that exposure to ammonia induced a significant reduction in histone and heterochromatin protein 1 expression. A reduction in cell viability was also noted. The ammonia-induced reduction of proliferative activity in these cultured astroglial cells seems to be due to a delay in the completion of the S phase provoked by the inhibition of chromatin protein synthesis.

## Introduction

Metabolic ammonia is the main causal agent of hepatic encephalopathy (HE) [1]. It has been suggested that ammonia might affect cerebral energy metabolism, neurotransmitter pathways, nitric oxide synthesis and signal transduction, and produce oxidative stress [2–4], but the exact pathological mechanisms underlying HE remain unknown.

Astroglial cells play a pivotal role in ammonia metabolism [5]. In fact, glutamine synthetase, the enzyme that detoxifies ammonia by condensing it with glutamate to form glutamine, is mainly found in astrocytes [6]. Astroglial dysfunction might, therefore, lead to nerve cell disease [7]. Many astroglial abnormalities have been reported in HE and hyperammonemia, with astroglial edema among the most prominent [8]. The effects of ammonia on astroglial proliferation, however, have been little documented. The many changes in cell physiology induced by ammonia might have an effect on the cell cycle (which is normally carefully regulated), and consequently on astroglial proliferation. However, it must be remembered that cell proliferation is reduced in this system in adult animals, even though the central nervous system possesses neural progenitor cells. *In vivo* studies showing ammonia-induced alterations of astroglial proliferative activity are very scarce [9, 10], but they suggest that proliferation is increased.

In our work on the role of astrocytes in HE, we use astroglial cell cultures as an *in vitro* model. In routine monitoring of these cultures it was noticed that, at confluence, the cells continued to proliferate, but were smaller. Ammonia-treated astroglial cells, however, showed no similar size reduction, perhaps because of a potentially lower proliferation rate. The aim of the present work was therefore to examine the effect of ammonia on the proliferative activity of cultivated astroglial cells. In order to determine when the effect(s) of ammonia occur, the proportions of cells in different phases of the cell cycle were noted, and BrdU incorporation and chromatin protein expression investigated.

## Materials and Methods

P0-P1 rats were anaesthetized with halothane to avoid unnecessary suffering and, after decapitation, the cerebral hemispheres dissected out. Astroglial cells, obtained as previously described [11], were grown in 75 cm^2^ flasks (primary cultures) containing DMEM medium (Gibco) supplemented with 10% fetal bovine serum (FBS) (Gibco) and an antibiotic/antimycotic solution (Gibco), at 37°C in a 5% CO_2_ atmosphere. Before confluence, the cells were detached with trypsin and reseeded (forming secondary cultures) in different multiwell plates (6, 24 and 96 wells) with FBS concentrations depending on the experiment (see below). The Wistar rats used to provide the astroglial cells were handled adhering to European Union Directive 63/2010/EC, Spanish law (Real Decreto 53/2013), and institutional guidelines on animal welfare prepared by the “Comité de Ética de Investigación y Experimentación Animal (Universidad de Alcalá)”. This study was approved by this committee and the sacrifice of the rats performed under its supervision.

Hyperammonemia was induced by adding 1, 3 or 5 mM NH_4_Cl to the culture medium. The hyperammonemic levels induced, which are pathophysiological in nature, are those most commonly employed in *in vitro* experiments. Given that NH_4_Cl completely dissociates, the final concentration of ammonia was the same as the NH_4_Cl concentration. Culture media were changed every three days, and new NH_4_Cl added to maintain stable ammonia concentrations.

### Cell number analysis

Detached astroglial cells were reseeded in 24-multiwell plates (12,000 cells/well) with 5% FBS. Three days later these cells were exposed to ammonia (1, 3 or 5 mM NH_4_Cl) for 1, 3 or 10 days. Both control and treated cells were then washed with PBS, detached with trypsin, washed again, and centrifuged (100 *g* for 5 min) in culture medium. After suspension in PBS, the cells were stained with trypan blue to identify those alive and dead; enumerating was performed using a Countess automatic cell counter (Invitrogen) and cell counting chamber slides. The experiment was performed in duplicate with six wells used for each duration and ammonia concentration.

### Viability: MTT assay

Astroglial cells were seeded (12,000 cells/well) and grown in 24-well plates, and the experiment begun three days after confluence was reached and the FBS content reduced to 5%. The cells were subjected to 1, 3 and 5 mM of NH_4_Cl for 1, 3, 5 and 10 days as above; this was performed in duplicate with 6 wells used for each duration and ammonia concentration. MTT (3-[4,5-dimethylthiazol-2-yl]-2,5-diphenyltetrazolium bromide) was used to assess cell viability. Formazan was solubilized with DMSO and colorimetric analysis performed using a LX800 microplate reader (Biotek Instruments) with 570/630 nm filters.

### Cell cycle analysis

The treatments and conditions were the same as above with the exception that the cells were reseeded in 6-multiwell plates (130,000 cells/well), and exposure to ammonia was for a full 10 days. After suspension in PBS, the cells were permeabilized and stained with propidium iodide. Their DNA content was measured using a FACSCalibur flow cytometer (Becton-Dickinson) employing a 488 nm argon ion laser as the excitation source. Cell cycle analysis was performed using Cylchred free software. The experiment was performed in triplicate, with six wells used for each ammonia concentration.

### Well DNA content

Cells were grown in 96-multiwell plates and exposed to ammonia as described above for 1, 5 or 10 days. After confluence the cells were fixed in formaldehyde (3% in PBS) for 20 min at room temperature and permeabilized using a solution of 0.1% Triton X-100 in PBS. A solution of DRAQ5 (diluted 1:2000) was used for DNA staining. After five 5 min washes and the complete removal of the wash solution, the plate was immediately scanned at 700 nm using an infrared imaging system (LICOR Biosciences). This experiment was performed only once, but using 20 wells for each duration and ammonia concentration.

### BrdU incorporation

The experimental conditions were the same as those described for cell number analysis, although the cells were grown over round coverslips to facilitate the quantification of BrdU incorporation. BrdU (Sigma) (1 μg/mL for 5 min) was added to the culture medium and incubated at 37°C. For the detection of BrdU, and following 4% paraformaldehyde fixation, the samples were incubated with the appropriate primary antibody (DAKO) (diluted 1:300) at 4°C overnight. An appropriate Cy3-labelled secondary antibody (Jackson) (diluted 1:1500 in blocking solution) was then added and incubation allowed to proceed for 1 h at room temperature. Nucleic acids were stained with DAPI. The stains were analyzed by conventional fluorescence microscopy (Zeiss) or confocal microscopy (Leica SP5). The number of positive cells was recorded as a percentage of the total number.

### Effect of ammonia on DNA polymerase

TaqDNA polymerase DNA replication efficiency was measured in real-time polymerase chain reactions (PCR) using Ct as a measure of amplification. Peripheral blood mononuclear cells from a donor were isolated by Ficoll gradient separation and their RNA isolated using the Ribozol^TM^ Plus RNA Purification Kit (Amresco). RNA concentrations were measured using a Quawell Q5000 spectrophotometer (Quawell). RNA integrity was verified using the Agilent RNA 6000 Nano Kit and employing a 2100 Bioanalyzer (Agilent). cDNA was generated from 1 μg of total RNA using the High Capacity cDNA Archive Kit (Life Technologies). The final reaction volume was 20 μL. Real-time PCR was performed in triplicate in a MicroAmpTM Fast 96-well reaction plate (0.1 mL) (Life Technologies) using 2 μL/well of a 1:10 dilution of cDNA plus 0.04 μM of *GAPDH* forward (5’-AGC CAC ATC GCT CAG ACA C-3’) and reverse (5’-GCC CAA TAC GAC CAA ATC C-3’) primers and 1× SYBR Green PCR Master Mix (Roche Applied Science) (total reaction volume 20 μL). PCR reactions were run at increasing concentrations (1, 3 and 5 mM) of NH_4_Cl. A control without NH_4_Cl or glutamine was used for comparisons. PCR reactions were run in a StepOne Plus^TM^ Real-Time PCR System (Life Technologies). ROX-normalized Ct values for each condition were compared using StepOne^TM^ Software v.2.3 (Life Technologies).

### iTRAQ protein analysis

To obtain sufficient protein for analysis, cultures were raised in 75 cm^2^ flasks—three for every condition combination studied (1, 3, 5 and 10 days, with 1, 3 or 5 mM NH_4_Cl). The experiment, performed in duplicate, began three days after confluence was reached and the FBS content had been reduced to 5%. Cells were detached and processed according to the Calbiochem Complete Proteo Extract Kit User’s Guide, and maintained at -80°C until use.

Cell pellets were dissolved in lysis buffer (8 M urea, 2 M thiourea, 5% CHAPS, 2 mM TCEP-HCl and protease inhibitor) and the cells subjected to ultrasonication (10 pulses at low amplitude) on ice. The lysed product was centrifuged at 20,000 *g* for 10 min at 4°C, and the supernatant containing the solubilized proteins later used in iTRAQ experiments. The total protein concentration was determined using the Pierce 660 nm Protein Assay Kit (Thermo). For digestion, 40 ug of the protein produced by each treatment were precipitated by the methanol/chloroform method. Protein pellets were resuspended and denatured in 20 μl of 7 M urea/2M thiourea/100 mM TEAB, pH 7.5, and reduced with 1 μL of 50 mM Tris(2-carboxyethyl) phosphine (TCEP, AB SCIEX), pH 8.0, at 37°C for 60 min. This was followed by the addition of 2 μL of 200 mM cysteine-blocking reagent (methyl methanethiosulfonate (MMTS) (Pierce) for 10 min at room temperature. Samples were diluted to 140 μL with 25 mM TEAB to reduce the urea/thiourea concentration. Digestions were initiated by adding 2 μL (1 μg/μL) sequence grade-modified trypsin (Sigma-Aldrich) to each sample in the ratio 1:20 (w/w); the reactions were incubated at 37°C overnight on a shaker. The digested samples were then labeled with different iTRAQ tags as follows: control, iTRAQ 114 or 115; celastrol-treated, iTRAQ 116 or 117. The samples were then pooled and dried and the peptides further labeled using the iTRAQ 4-plex Kit (AB SCIEX) according to the manufacturer's instructions. After labeling, the samples were pooled, dried and desalted using a SEP-PAK C18 Cartridge (Waters). Finally, the cleaned tryptic peptides were evaporated to dryness and stored at -20°C until analysis.

A 2 μg aliquot of these pooled, cleaned, tryptic peptides was subjected to 2D-nano LC ESI-MSMS analysis using a nano liquid chromatography system (Eksigent Technologies nanoLC Ultra 1D plus, AB SCIEX) coupled to high speed Triple TOF 5600 mass spectrometer (AB SCIEX) with a duo spray ionization source. The analytical column used was a silica-based reversed phase column (C18 ChromXP, 75 μm × 15 cm, 3 μm particle size, 120 Å pore size) (Eksigent Technologies, AB SCIEX). The trap column was also a C18 ChromXP model, in line with the analytical column. The loading pump delivered a solution of 0.1% formic acid in water at 2 μL/min. The nano-pump provided a flow rate of 300 nL/min and was operated under gradient elution conditions using 0.1% formic acid in water as mobile phase A, and 0.1% formic acid in acetonitrile as mobile phase B. Peptides with iTRAQ labels were separated using a 200 min gradient ranging from 2% to 90% mobile phase B (mobile phase A: 2% acetonitrile, 0.1% formic acid; mobile phase B: 100% acetonitrile, 0.1% formic acid). The injection volume was 5 μL.

Data were acquired using a TripleTOF 5600 System (AB SCIEX) with the following settings: ion spray voltage floating (ISVF) 2800 V, curtain gas (CUR) 20 l/h, interface heater temperature (IHT) 150°C, ion source gas 1 (GS1) 20 l/h, declustering potential (DP) 85 V. All data were collected in information-dependent acquisition (IDA) mode using Analyst TF 1.5 software (AB SCIEX). A 0.25 s MS survey scan was performed in the mass range 350–1250 Da, followed by 30 MS/MS scans of 150 ms in the mass range 100–1800 (total cycle time: 4.04 s). Switching criteria were set to ions with a mass/charge ratio (m/z) of >350 but <1250, with a charge state of 2–5 and an abundance threshold of more than 70 counts (cps). Former target ions were excluded for 20 s. The IDA rolling collision energy (CE) parameters script option was used for automatically controlling the CE.

MS and MS/MS data obtained for pooled samples were processed using Analyst® TF 1.5.1 software (AB SCIEX). Raw data file conversion tools generated mgf files which were compared against the *Homo sapiens* database (which contains 40,530 protein-coding genes and their corresponding reversed entries), using the Mascot Server v. 2.5.0 (Matrix Science). The search parameters were: enzyme, trypsin; allowed missed cleavages, 1; fixed modifications, iTRAQ4plex (N-term and K) and beta-methylthiolation of cysteine; variable modifications, oxidation of methionine. Peptide mass tolerance was set at ± 25 ppm for precursors and 0.05 Da for fragment masses. The confidence interval for protein identification was set to ≥95% (p<0.05); only peptides with an individual ion score above the 1% false discovery rate (FDR) threshold were considered correctly identified. Only proteins with at least two quantified peptides were considered in quantitation. A 5% quantitation FDR threshold was used to consider proteins differentially expressed.

### Statistical analysis

One-way ANOVA followed by a Bonferroni post-hoc comparison for pairwise tests was used to compare the measured variables between control and treatment cultures. Only significant changes between the control and ammonia-treated groups are included in the figures; significant changes between different ammonia-treated groups are not included.

## Results

**Fig 1:**
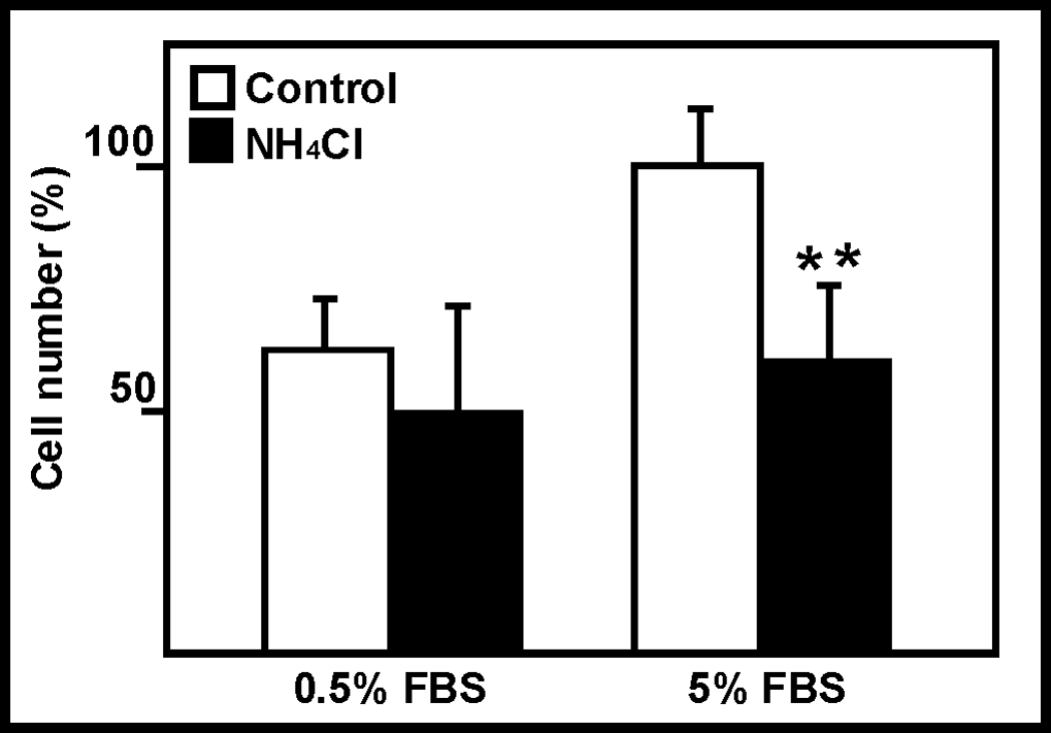
Effect of ammonia and serum on astroglial proliferation. Effect of ammonia (NH_4_Cl) exposure on astroglial proliferation was tested at two FBS concentrations (0.5 and 5%). **p<0.01. The percentage value is calculated taking the value for the 5% FBS control as 100% (1.65 x 10^5^ cells). Columns and bars represent means and SDs.

To determine the optimum conditions for studying changes in the proliferative activity of the cells, a preliminary experiment was performed. It is logical to assume that cells growing under very low FBS content conditions might have a low proliferative rate, and that this might make it difficult to detect any effect of ammonia. Confluent astroglial cells were exposed to 5 mM NH_4_Cl for 10 days with either 0.5 or 5% FBS. Fig 1 shows that ammonia affects astroglial proliferation, but this effect was only clear for the cells cultured with 5% FBS: a significant 40% reduction (p<0.01) was detected with 5% FBS, but only a non-significant 20% reduction was observed in cells growing with 0.5% FBS.

### Cell number analysis and viability

**Fig 2:**
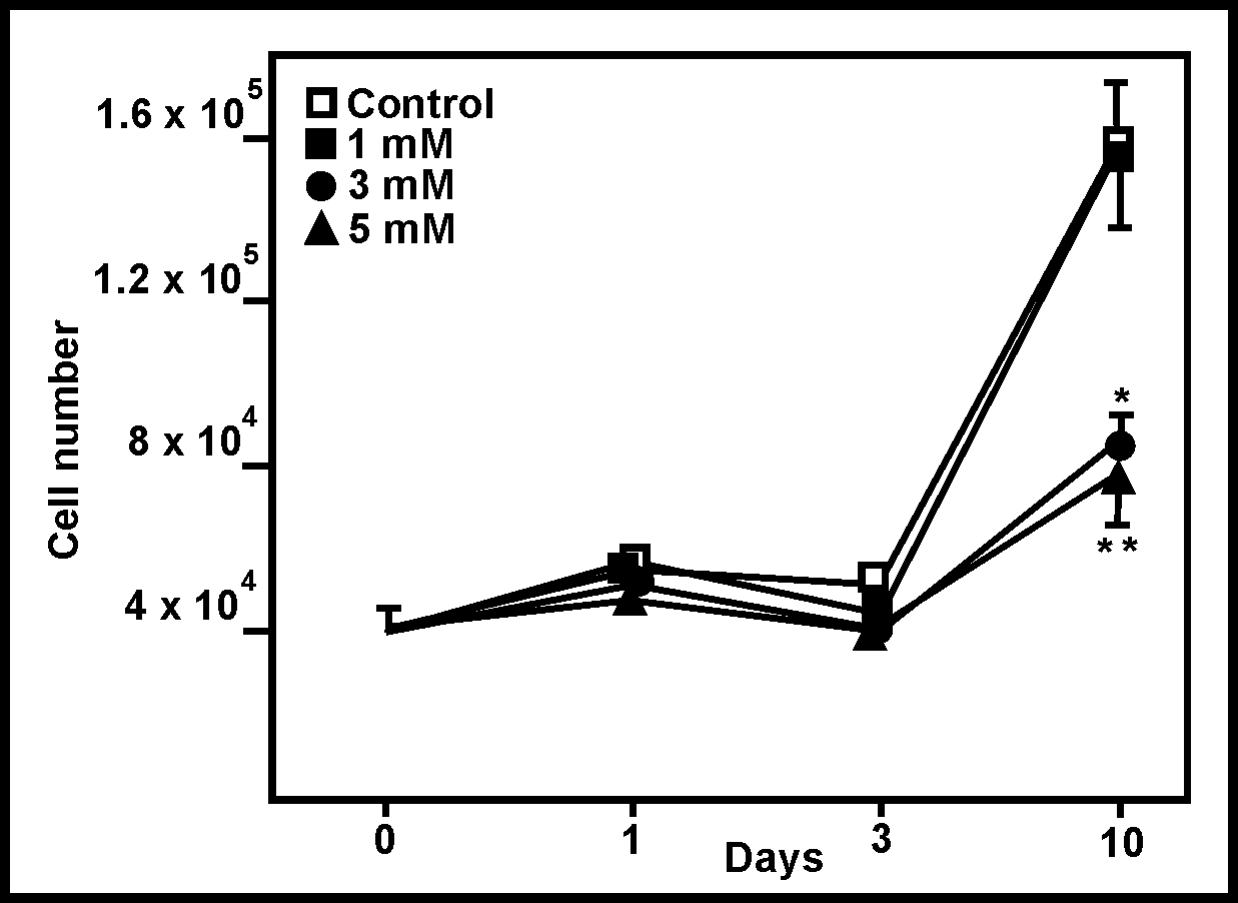
Effect of ammonia on astroglial proliferation. Change in astroglial cell number after 10 days of exposure to ammonia. Standard deviations are not shown for 1 and 3 days since differences between the control and ammonia-treated cells were not significant at these times. *p<0.05, **p<0.01.

Exposure to ammonia induced a dose- and time-dependent reduction in cell number. Clear, significant reductions were observed after 10 days in the 3 and 5 mM ammonia treatments (44.8 and 50.4% respectively; p<0.01) (Fig 2). These reductions were not due to an increase in the number of dead cells; their number was small and very similar under all experimental condition (data not shown).

**Fig 3:**
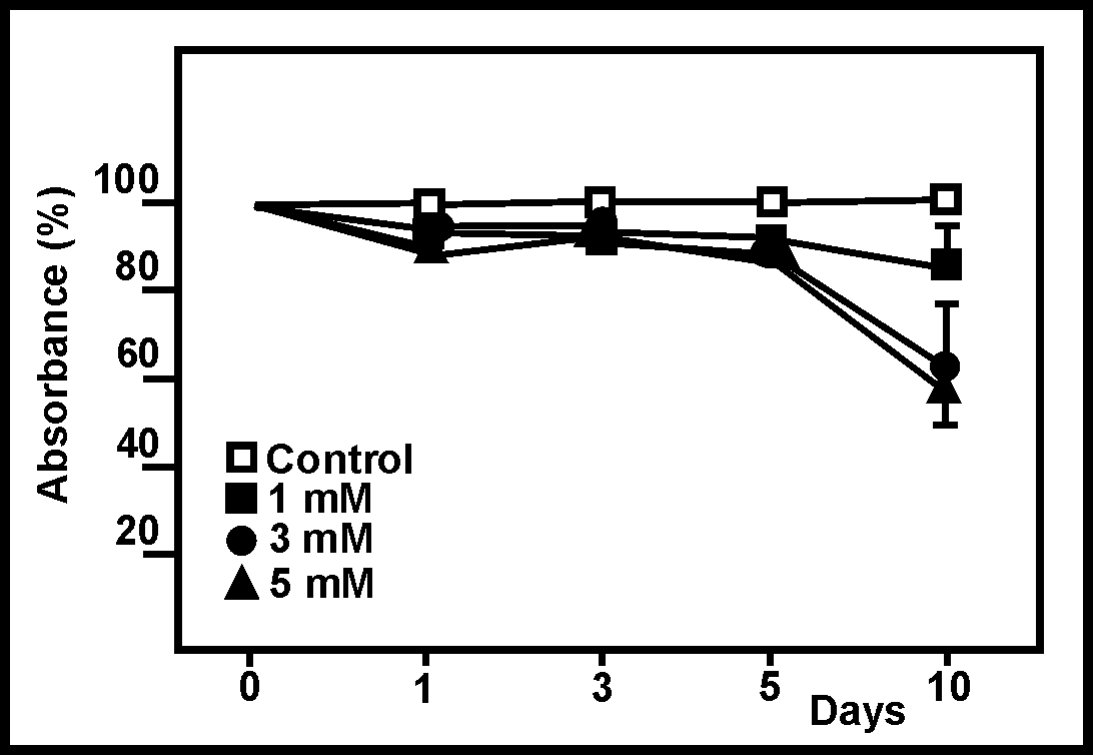
Effect of ammonia on viability of astroglial cells. MTT viability assay of astroglial cells exposed to ammonia. Points and bars represent means and SDs. No SD values are included for 1, 3 and 5 days since they were <10% different to the mean.

The MTT assay showed ammonia to affect cell viability in a dose- and time-dependent manner (Fig 3). A 40% reduction in viability was detected after 10 days of exposure to 5 mM ammonia, a reduction reflected in the cell count analysis. Indeed, a strong correlation was seen between the cell count (Fig 2) and viability data (Fig 3) (r = 0.933, p = 0.51x10^-4^).

### Cell cycle analysis

**Fig 4:**
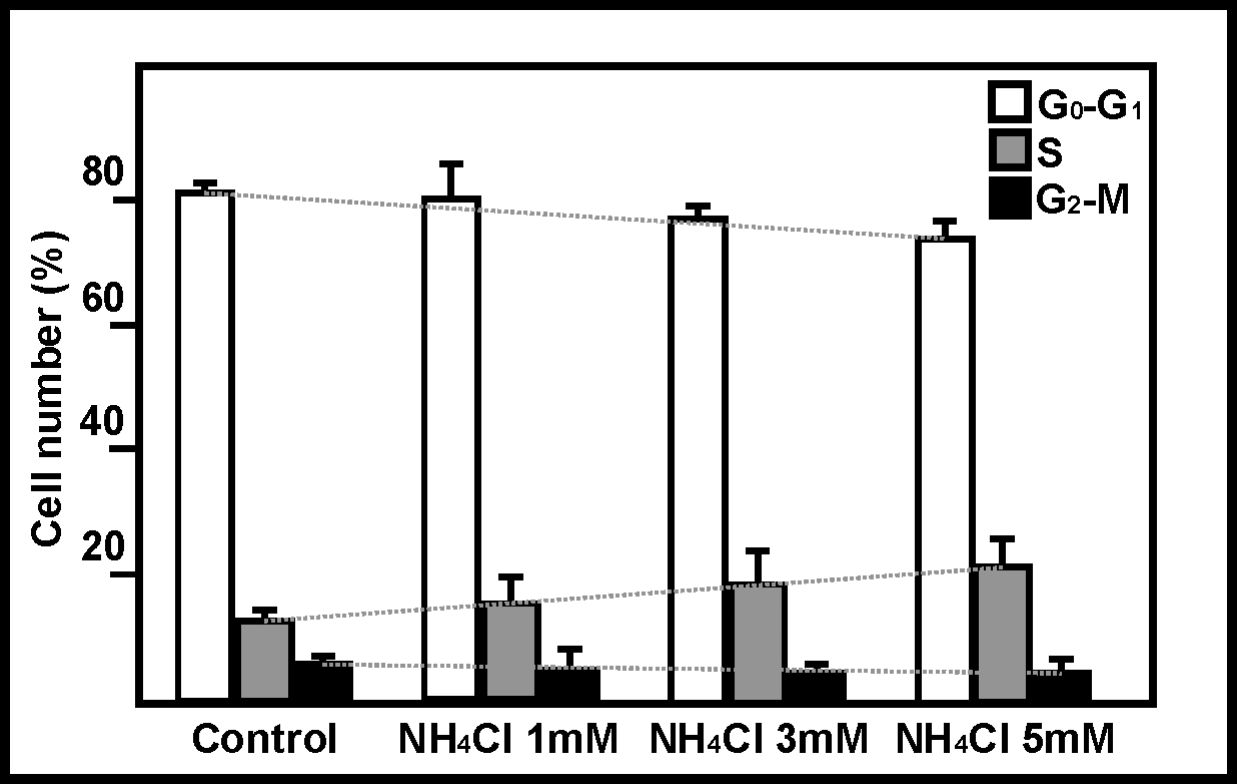
Effect of ammonia on cell cycle phases. Cell cycle analysis after 10 days of exposure to 1, 3 and 5 mM ammonia. The dotted lines are included to indicate the trends for the cell cycle phases. Columns and bars represent means and SDs. The percentages of cells in the phases considered (G_0_-G_1_, S and G_2_-M) are included for every experimental condition; it should be noted that the sum of the three phases is 100% (10^4^ cells).

No significant differences were observed between treatments in terms of the number of cells in the G_2_-M phase. However, a clear increase in the number of cells in the S phase was detected (reaching 9% with the 5 mM ammonia treatment), with a concomitant reduction in the number of cells in the G_0_-G_1_ phase (Fig 4). Using the algorithm N = N_o_ x 2^t/g^ (where N and N_o_ are the final and initial cell numbers, t the duration of treatment, and g the duration of the cell cycle), and taking into account (1) a cell cycle length 20 h [12], (2) 10 days of exposure, and (3) that ammonia induces a 10% time delay in the completion of the cell cycle, a theoretical reduction of 46% is obtained. This figure lies between the 40% and 50% reductions detected in cell count analysis.

### Well DNA content

**Fig 5:**
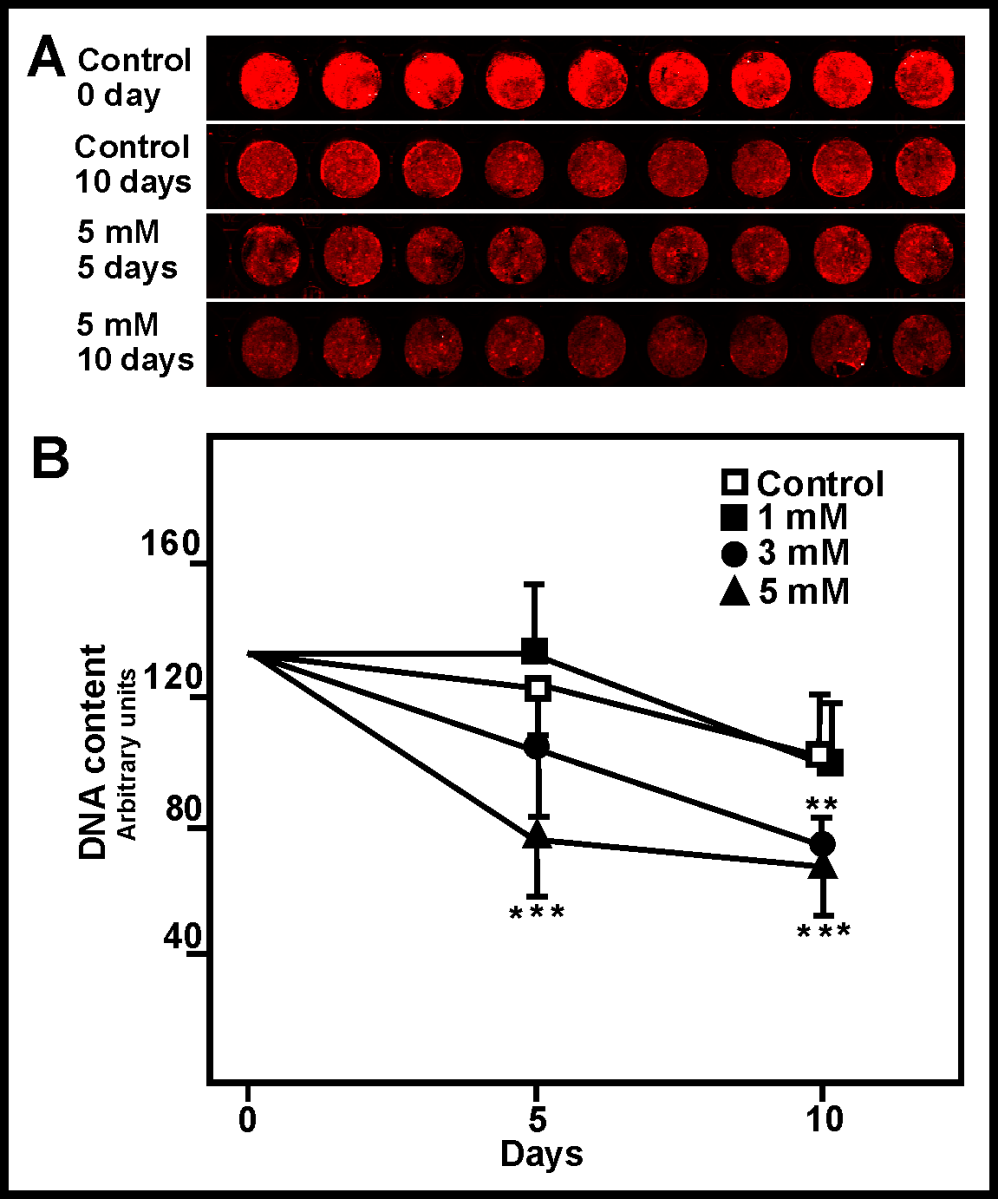
Effect of ammonia on DNA content. (A) Four rows of the in-cell DNA content analysis plate; note that ammonia exposure reduced the fluorescence signal. The row shows only nine results since the external wells were not seeded and the control point without fluorescent dye is not included. (B) Plot showing the change in well DNA content after ammonia exposure. Points and bars represent means and SDs. **p<0.05, ***p<0.01.

A lower cell proliferation rate leads to a reduction in cell number, which should be associated with a lower well DNA content. This was measured using an in-cell based assay to avoid protocols including cell fragmentation and DNA extraction. In general, the well DNA content was reduced when cells were treated with ammonia, with significant changes observed after 5 days of exposure to the 5 mM concentration, and after 10 days of exposure to the 3 mM concentration (Fig 5).

### BrdU incorporation

**Fig 6:**
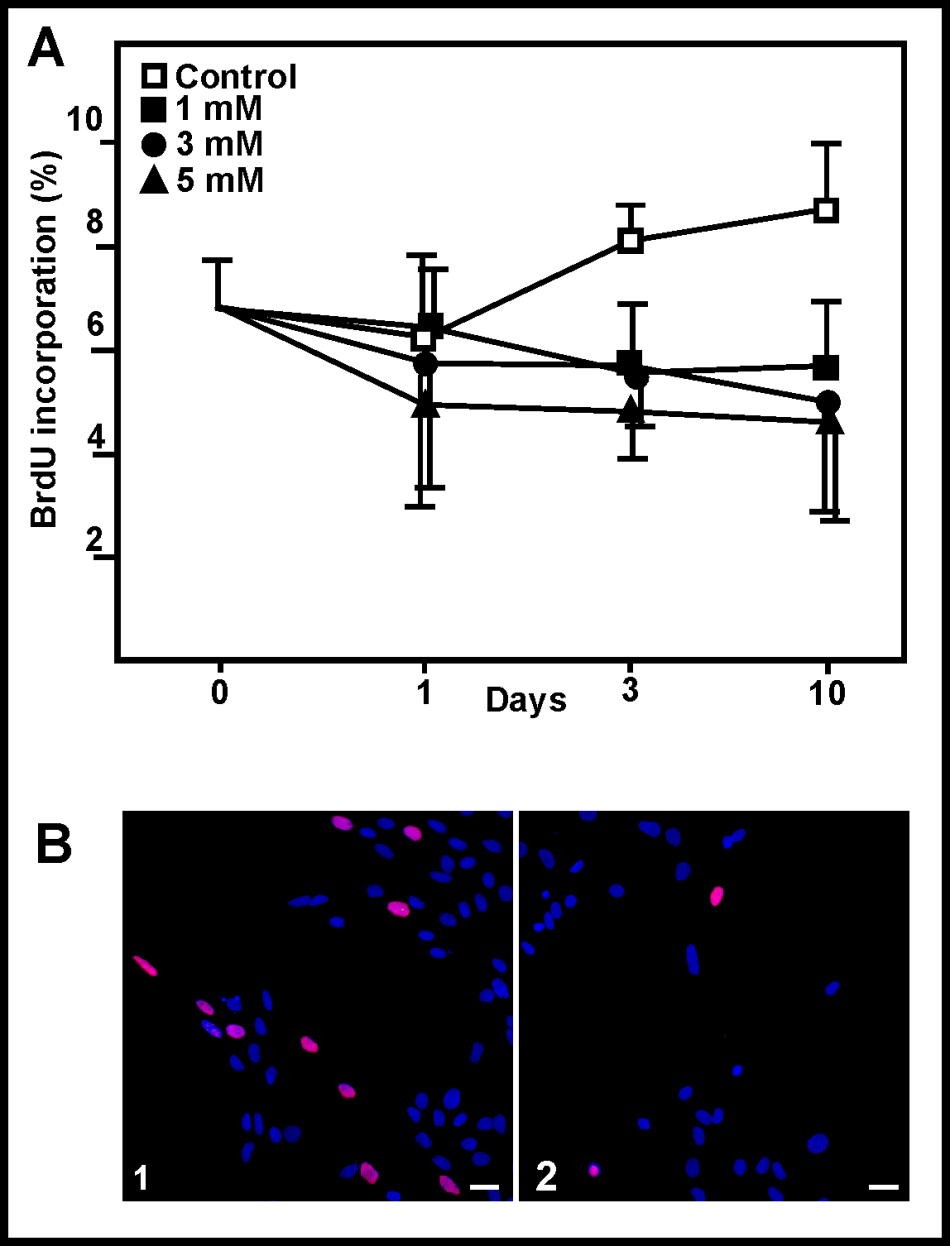
Effect of ammonia on BrdU incorporation. (A) BrdU incorporation by astroglial cells following exposure to ammonia. Points and bars represent means and SD. No significant changes were observed. (B) Cultured cells showing BrdU incorporation (red): (1) 1 day control, (2) 3 days after exposure to 5 mM ammonia. Scale bar: 10 μm.

The delay in the completion of the S phase was examined by BrdU incorporation. BrdU is incorporated into newly synthesized DNA in the place of thymidine, and is very easy to detect; slower DNA synthesis should thus be revealed by reduced BrdU incorporation. BrdU was incorporated to a lesser extent by ammonia-treated than by control cells (Fig 6). At 10 days, the 5 mM ammonia-treated cells incorporated 45.5% less BrdU than did controls, in line with the reduction recorded in cell count analysis.

### Effect of ammonia on DNA polymerase

**Fig 7:**
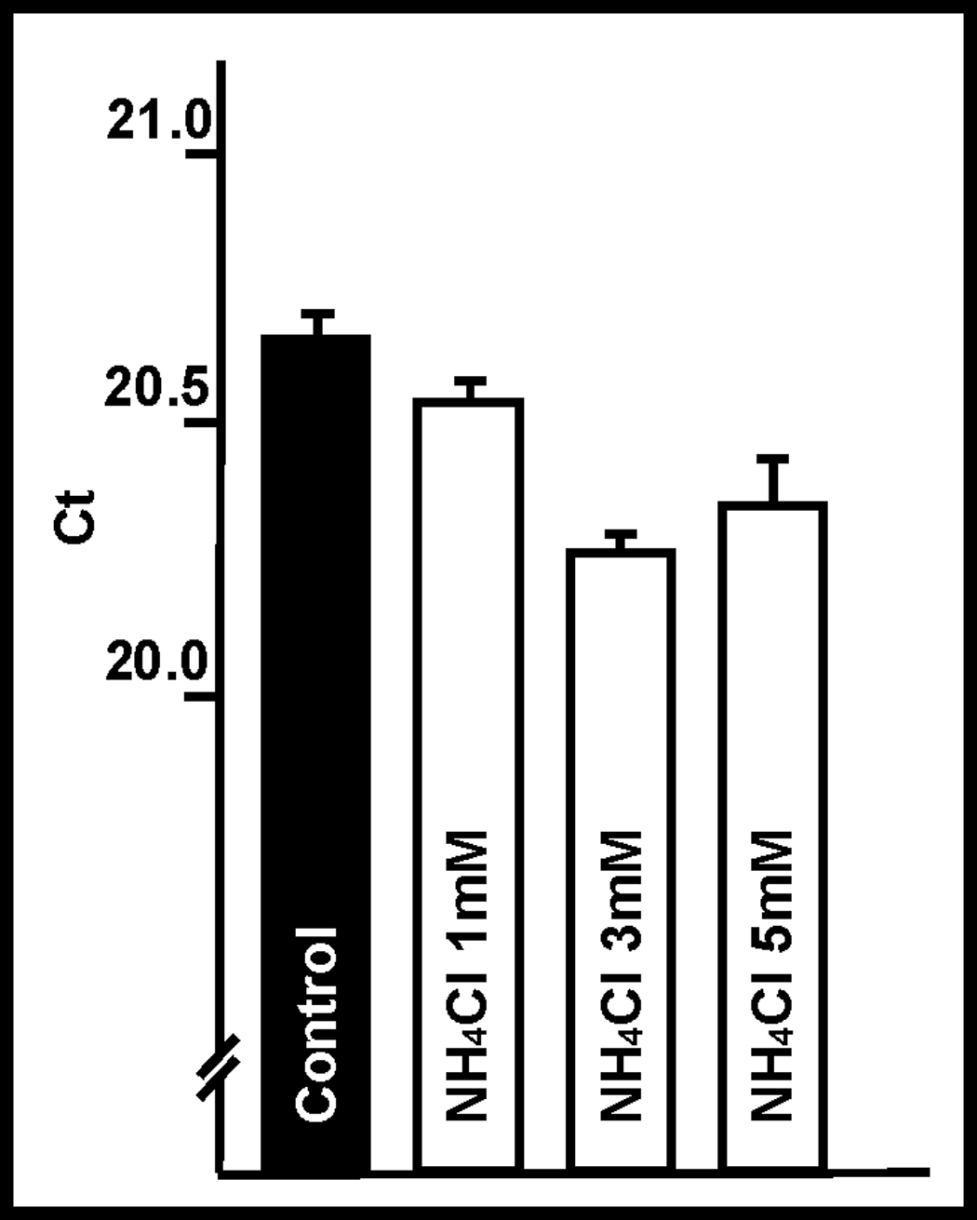
Effect of ammonia on DNA polymerase. Effect of ammonia (NH_4_Cl) on the activity of DNA polymerase, analyzed via real-time PCR. Note that the Ct variation ranges between 20 and 21. Columns and bars represent means and SDs. No significant changes were observed.

To determine whether the delay in DNA synthesis was due to an ammonia dysfunction involving DNA polymerase, real-time PCR analysis starting with the same quantity of cDNA was performed with or without ammonia in the reaction medium. Fig 7 shows that ammonia had no significant effect on DNA amplification or, consequently, on DNA polymerase function. Given that a difference of 1 unit in Ct corresponds to a 2x-fold expression change, the differences observed under the different ammonia conditions are extremely small.

### iTRAQ protein analysis

Table 1 shows that the expression of histones H1, H2A, H2B, H3 and H4, and of heterochromatin protein 1 (HP1, a non-histone chromatin protein), was reduced by exposure to ammonia in a dose-dependent manner.

**Table 1:** iTRAQ analysis of histones and HP1 expression.

		H1	H2A	H2B	H3	H4	HP1
**1 mM NH**_**4**_ **Cl**	p-value	0.012	0.038	0.402	0.05	0.007	0.11
	z-score	-2.50	-2.06	-0.83	-1.95	-2.69	-1.59
**3 mM NH**_**4**_ **Cl**	p-value	0.05	0.005	0.067	0.028	0.009	0.022
	z-score	-1.88	-2.60	-1.82	-2.18	-2.58	-2.28
**5 mM NH**_**4**_ **Cl**	p-value	1.3 10^−5^	4.2 10^−5^	0.03	6.5 10^−4^	1.1 10^−4^	0.005
	z-score	-4.34	-3.00	-2.08	-3.40	-3.86	-2.79

Change in p-value and z-score for histones (H1, H2A, H2B, H 3 and H4) and HP1 determined by iTRAQ analysis of astroglial cells exposed to ammonia for 5 days.

## Discussion

The effect of ammonia on cell proliferation has been studied in enterocytes, kidney epithelial cells and nerve cells, but information regarding its effect on cell proliferation in astroglia is very scarce, and what there is can be contradictory. In general, ammonia inhibits cell proliferation; this has been reported for human gastric cancer cells (HGC-27, MKN-1 and MKN-45) [13, 14], human colon adenocarcinoma cells (HF-29) [15], and human prostatic cancer cells (DU-145) [16]. However, contradictory results were obtained in the first autoradiographic studies: a dose-dependent depression of cell replication was seen in cultured kidney cells [17] yet no influence on proliferation was observed in rat enterocytes (IEL-6 line) [18]. The first studies on the effect of ammonia in *in vivo* models suggested there to be an increase in astroglial proliferation [9,10]. In fact, DNA synthesis has been shown in Alzheimer type II astrocytes, a cell type that appears in HE [19]. However, the results of the present work show that ammonia reduces the proliferative activity of astroglial cells in culture. Similar inhibition has been reported for astrocytoma cells [20] and cultured astrocytes [21]. The fact that increased and decreased proliferative activity has been described in *in vivo* and *in vitro* models respectively may reflect differences in these models themselves; moreover, it should be remembered that *in vitro* models cannot fully reproduce HE. It is well known that astroglial cells proliferate in the adult central nervous system and that this proliferative activity may be involved in the development of different cognitive functions. HE causes, among other neurological disorders, the impairment of memory formation [22], and astrocytes have been clearly involved in establishing memory [23]. Thus, it might be that a reduction in astroglial proliferation is involved in the memory and cognitive alterations seen in HE [24, 25].

A number of mechanisms have been proposed to explain the inhibition of cell proliferation from a cell cycle perspective. The accumulation of cells in the S [14] or G_2_-M [26] phases in ammonia-treated cells has been reported, but so has the repression of proliferation with no changes in the distribution of cells in the different phases of the cell cycle [15]. The inhibitory effect of ammonia on the synthesis of polyamines (metabolites needed for cell growth) has been proposed responsible for the latter. The present results, and those of Matsui et al. [14] in enterocytes, clearly show that exposure to ammonia causes astroglial cells to become delayed in the S phase. This might increase the length of the cell cycle by 10%, enough to explain the inhibition of proliferation observed in the present work. The possibility that ammonia might inhibit entry into the cell cycle has also been suggested; indeed, the p53-mediated enhanced transcription of cell cycle inhibitory genes (GADD45α and p21) in the presence of ammonia has recently been demonstrated [21]. The inhibition of cell cycle entry by ammonia [21], and the induction of cell cycle delay (present results) are not mutually exclusive processes. On the contrary, they should be considered complementary strategies aimed at reducing astroglial proliferation.

The synthesis of chromatin is a pivotal event in the S phase. Chromatin is composed of DNA and specific proteins, and the synthesis of both is finely regulated; in fact, an imbalanced synthesis compromises chromosome replication. The present results show that ammonia inhibits DNA synthesis, as indicated by the reduced DNA content and BrdU incorporation observed in the ammonia-treated cells. A similar inhibitory effect of ammonia on DNA synthesis has been reported in other studies [14, 17, 27]. Neither BrdU incorporation nor mitotic figures were observed in the liver of rats in an *in vivo* model of fulminant hepatic failure associated with hyperammonemia [28]. However, DNA synthesis and mitotic divisions have been described in Alzheimer type II astrocytes, cells associated with HE [19]. To determine whether the inhibition of DNA synthesis was due to an effect of ammonia and/or glutamine on DNA polymerase, the functioning of this enzyme under high ammonia concentrations was tested using PCR. No significant changes in the enzyme’s functioning were seen; it seems that DNA polymerase is not affected by ammonia.

A reduction in DNA synthesis should be associated with an inhibition in chromatin protein expression. The present results are the first to reveal an inhibitory effect of ammonia on the expression of histones H1, H2A, H2B, H3 and H4, and HP1. The present results cannot, of course, reveal whether the inhibition of DNA synthesis is the cause or the effect of the inhibition of chromatin protein expression. However, it is well known that most histone proteins are produced during the S phase, and that their synthesis depends on DNA replication. In fact, these histones are known as replication-dependent proteins [29]. Thus, the inhibition of DNA synthesis by ammonia ought to inhibit histone synthesis. HP1 is a non-histone chromosomal protein associated with heterochromatin; it is essential for maintaining the active transcription of genes involved in cell cycle progression [30].

It is well known that energy metabolism influences the progression of the cell cycle, and that ammonia affects the metabolic status of the cell. Thus, the effect of ammonia on astroglial proliferation might be due to an energy imbalance. However, the results show that exposure to ammonia greatly reduced cell viability, and that this correlated strongly with the reduction in cell number. This suggests that the loss of viability is almost exclusively due to the reduction in cell number and not to alterations in energy metabolism. The possibility that the loss of viability is owed to cell death can be ruled out since the number of dead cells was very low and similar under all experimental conditions. Although a clear inhibitory effect of ammonia leading to mitochondrial dysfunction has been observed in different studies [31, 32], the effects of ammonia on cerebral oxidative energy metabolism are heterogeneous [33]. Certainly, the role of astroglial cells in cerebral oxidative metabolism remains somewhat unclear. Mitochondrial dysfunction induced by changes in mitochondrial permeability transition [34], and in the malate-aspartate shuttle [35], have been observed in *in vitro* studies, but no changes in astroglial oxidative metabolism have been seen in *in vivo* studies [36]. The present iTRAQ results confirm that ammonia induces alterations in astroglial energy metabolism, but the response is positively regulated, as suggested by the significant increase detected in many mitochondrial proteins, including ADP/ATP translocase, mitochondrial import receptor subunit TOM6, and ATP synthetase subunit δ (z-score 3.11, 2,54, and 1.8 respectively). The possibility that these changes represent an adaptive response to ammonia-induced alterations in mitochondrial metabolism of astrocytes needs to be investigated.

### Conclusions

Ammonia induces a dose- and time-dependent reduction in the proliferative activity of astroglial cells in culture. The cell cycle phase analysis and BrdU incorporation results show this reduction to be due to an accumulation of cells in the S phase, as corroborated by a reduced well DNA content and the strong inhibition of the expression of histones and HP1. The interference of energy status by ammonia as a reason for reduced proliferation is not supported. An effect of ammonia and glutamine on DNA polymerase, the enzyme that replicates DNA, does not seem probable, although it cannot be completely ruled out. Further studies are required to determine the precise mechanisms by which ammonia affects DNA synthesis.

## Supporting Information

S1 Checklist ARRIVE checklist. (DOCX)

## References

[1] Butterworth RF, Giguère JF, Michaud J, Lavoie J, Layrargues GP. Ammonia: key factor in the pathogenesis of hepatic encephalopathy. Neurochem Pathol. 1987; 6: 1–12. 10.1007/BF02833598 3306479

[2] Bosoi CR, Rose CF. Identifying the direct effects of ammonia on the brain. Metab Brain Dis. 2009; 24: 95–102. 10.1007/s11011-008-9112-7 19104924

[3] Häussinger D, Schliess F. Pathogenetic mechanisms of hepatic encephalopathy. Gut. 2008; 57: 1156–1165. 10.1136/gut.2007.122176 18628377

[4] Ott P, Vilstrup H. Cerebral effects of ammonia in liver disease: current hypotheses. Metab Brain Dis. 2014; 29: 901–911. 10.1007/s11011-014-9494-7 24488230

[5] Norenberg MD. The role of astrocytes in hepatic encephalopathy. Neurochem Pathol. 1987; 6: 13–33. 3306480 10.1007/BF02833599

[6] Norenberg MD, Martínez-Hernández A. Fine structural localization of glutamine synthetase in astrocytes of rat brain. Brain Res. 1979; 161: 303–310. 31966 10.1016/0006-8993(79)90071-4

[7] Albrecht J. Astrocytes in ammonia neurotoxicity: A target, a mediator and a shield In: Aschner M, Costa LG, editors. The Role of Glia in Neurotoxicity. Boca Raton: CRC Press; 2005 pp. 329–342.

[8] Rama Rao KV, Norenberg MD. Aquaporin-4 in hepatic encephalopathy. Metab Brain Dis. 2007; 22: 265–275. 17879149 10.1007/s11011-007-9063-4

[9] Norenberg MD, Lapham LW, Eastland ME, May AG. Division of protoplasmic astrocytes in acute experimental hepatic encephalopathy. An electron microscopy study. Am J Pathol. 1972; 67: 403–408. 5021108 PMC2032596

[10] Diemer NH, Laursen H. Glial cell reactions in rats with hyperammoniemia induced by urease or porto-caval anastomosis. Acta Neurol Scand. 1977; 55: 425–442. 878834 10.1111/j.1600-0404.1977.tb07623.x

[11] Bodega G, Suárez I, López-Fernández LA, Almonacid L, Zaballos A, Fernández B. Possible implication of ciliary neurotrophic factor (CNTF) and β-synuclein in the ammonia effect on rat astroglial cells in culture. A study using DNA and protein microarrays. Neurochem Int. 2006; 48: 729–738. 16483693 10.1016/j.neuint.2005.12.014

[12] Knapp PE. The cell cycle of glial cells grown in vitro: an immunocytochemical method of analysis. J Histochem Cytochem. 1992; 40: 1405–1411. 1506676 10.1177/40.9.1506676

[13] Matsui T, Matuskawa Y, Sakai T, Nakamura K, Aoike A, Kawai K. Effect of ammonia on cell-cycle progression of human gastric cancer cells. Eur J Gastroen Hepatol. 1995; 7 (Suppl. 1): S79–S81. 8574744

[14] Matsui T, Matuskawa Y, Sakai T, Nakamura K, Aoike A, Kawai K. Ammonia inhibits proliferation and cell cycle progression at S-phase in human gastric cells. Digest Dis Sci. 1997; 42: 1394–1399. 9246035 10.1023/a:1018837920769

[15] Mouillé B, Delpal S, Mayeur C, Blachier F. Inhibition of human colon carcinoma cell growth by ammonia: a non-cytotoxic process associated with polyamine synthesis reduction. BBA-Gen Subjects. 2003; 1624: 88–97. 10.1016/j.bbagen.2003.09.014 14642818

[16] Won JH, Parekattil SJ, Davidson SD, Luddy JS, Choudhury MS, Mallouh C, et al Ammonium-chloride-induced prostatic hypertrophy in vitro: urinary ammonia as a potential risk factor for benign prostatic hyperplasia. Urol Res. 1999; 27: 376–381. 10550527 10.1007/s002400050166

[17] Rabkin R, Palathumpat M, Tsao T. Ammonium chloride alters renal tubular cell growth and protein turnover. Lab Invest. 1993; 68: 427–438. 8479151

[18] Zachrisson K, Midtvedt T, Uribe A. The in vitro effect of ammonia on DNA synthesis in rat enterocytes. Eur J Gastroent Hepatol. 1994; 6: 951–954.

[19] Brumback RA, Lapham LW. DNA synthesis in Alzheimer type II astrocytosis. The question of astrocytic proliferation and mitosis in experimentally induced hepatic encephalopathy. Arch Neurol. 1989; 46: 845–848. 2757524 10.1001/archneur.1989.00520440027016

[20] Hillmann P, Köse M, Söhl K, Müller CE. Ammonium-induced calcium mobilization in 1321N1 astrocytoma cells. Toxicol Appl Pharm. 2008; 227: 36–47. 10.1016/j.taap.2007.10.012 18061226

[21] Görg B, Karababa A, Shafigulina A, Bidmon HJ, Häussinger D. Ammonia-induced senescence in cultured rat astrocytes and in human cerebral cortex in hepatic encephalopathy. Glia. 2015; 63: 37–50. 10.1002/glia.22731 25092802

[22] Leke R, Oliveira DL, Forgiarini LF, Escobar TD, Hammes TO, Meyer FS, et al Impairment of short term memory in rats with hepatic encephalopathy due to bile duct ligation. Metab Brain Dis. 2013; 28: 187–192. 10.1007/s11011-012-9347-1 23111918

[23] Santello M, Volterra A. Astrocytes as aide-mémoires. Nature. 2010; 463: 169–170. 10.1038/463169a 20075911

[24] Weissenborn K, Giewekemeyer K, Heidenreich S, Bokemeyer M, Berding G, Ahl B. Attention, memory, and cognitive function in hepatic encephalopathy. Metab Brain Dis. 2005; 20: 359–367. 16382346 10.1007/s11011-005-7919-z

[25] Méndez M, Méndez-López M, López L, Aller MA, Arias J, Arias JL. Associative learning deficit in two experimental models of hepatic encephalopathy. Behav Brain Res. 2009; 198: 346–351. 10.1016/j.bbr.2008.11.015 19056427

[26] Renau-Piqueras J, O’Connor JE, Báguena-Cervellera R, Grisolía S. Ammonium chloride-induced alterations in growth kinetics and ultrastructure of murine neuroblastoma cells. A flow cytometric and stereologic analysis. Virchows Arch B Cell Pathol Incl Mol Pathol. 1986; 50: 271–283. 2870578 10.1007/BF02889906

[27] Franch HA. Modification of the epidermal growth factor response by ammonia in renal cell hypertrophy. J Am Soc Nephrol.2000; 11: 1631–1638. 10966487 10.1681/ASN.V1191631

[28] Eguchi S, Kamlot A, Ljubimova J, Hewitt WR, Lebow LT, Demetriou AA, et al Fulminant hepatic failure in rats: survival and effect on blood chemistry and liver regeneration. Hepatology. 1996; 24: 1452–1459. 8938180 10.1002/hep.510240626

[29] Rattray AMJ, Müller B. The control of histone gene expression. Biochem Soc Trans. 2013; 40: 880–885. 10.1042/BST20120065 22817752

[30] De Lucia F, Ni J-Q, Vaillant C, Sun F-L. HP1 modulates the transcription of cell cycle regulators in *Drosophila melanogaster* . Nucl Acids Res. 2005; 33: 2852–2858. 15905474 10.1093/nar/gki584 PMC1131934

[31] Bjerring PN, Larsen FS. Changes in cerebral oxidative metabolism in patients with acute liver failure. Metab Brain Dis. 2013; 28: 179–182. 10.1007/s11011-012-9346-2 23099996

[32] Rama Rao KV, Norenberg MD. Cerebral energy metabolism in hepatic encephalopathy and hyperammonemia. Metab Brain Dis. 2001; 16: 67–78. 11726090 10.1023/a:1011666612822

[33] Schousboe A, Waagepetersen HS, Leke R, Bak LK. Effects of hyperammonemia on brain energy metabolism: controversial findings in vivo and in vitro. Metab Brain Dis. 2014; 29: 913–917. 10.1007/s11011-014-9513-8 24577633

[34] Rama Rao KV, Jayakumar AR, Norenberg DM. Ammonia neurotoxicity: role of the mitochondrial permeability transition. Metab Brain Dis. 2003; 18: 113–127. 12822830 10.1023/a:1023858902184

[35] Kala G, Hertz L. Ammonia effects on pyruvate/lactate production in astrocytes—Interaction with glutamate. Neurochem Int. 2005; 47: 4–12. 15890434 10.1016/j.neuint.2005.04.001

[36] Iversen P, Mouridsen K, Hansen MB, Jensen SB, Sørensen M, Bak LK, et al Oxidative metabolism of astrocytes is not reduced in hepatic encephalopathy: a PET study with [^11^C]acetate in humans. Front Neurosci. 2014; 8.353. 10.3389/fnins.2014.00353 PMC4217371 25404890

