# Supplementary material for: Ammonia Affects Astroglial Proliferation in Culture
Source: PLoS One. 2015 Sep 30;10(9):e0139619. doi: 10.1371/journal.pone.0139619 (PMC4589356; doi:10.1371/journal.pone.0139619)

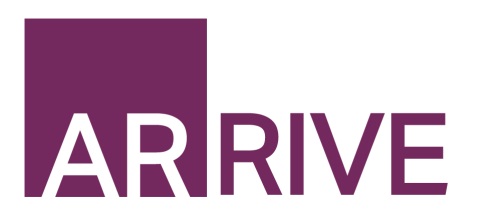


The ARRIVE Guidelines Checklist

**Ammonia affects astroglial proliferation in culture**

Guillermo Bodega^1*^, Berta Segura^2^, Sergio Ciordia^3^, María del Carmen Mena^3^, Luis Andrés López-Fernández^4^, María Isabel García^4^, Isabel Trabado^5^ and Isabel Suárez^1^

|  | | ITEM | RECOMMENDATION | Section/ Paragraph |
| --- | --- | --- | --- | --- |
| 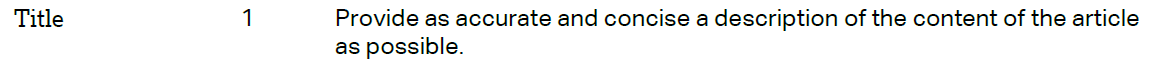 | | | Title |  |
| 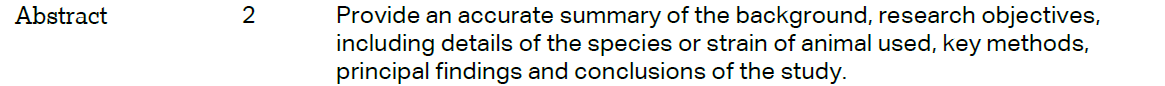 | | | Abstract |  |
| INTRODUCTION | | |  |  |
| 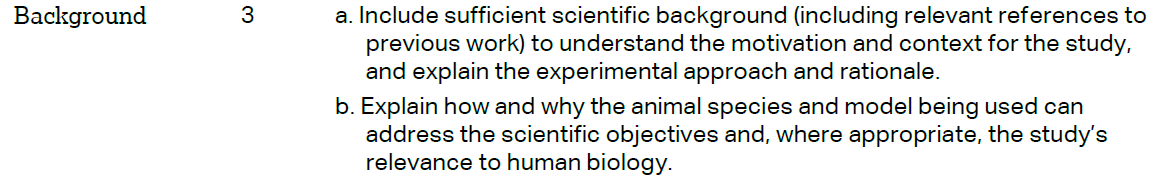 | | | Introduction  Paragraphs 1 and 2  Introduction  Paragraph 3 |  |
| 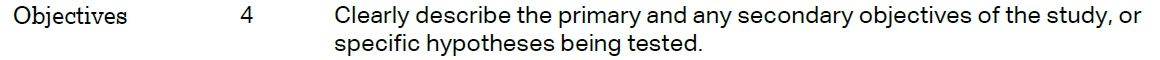 | | | Introduction  Paragraph 3 |  |
| METHODS | | |  |  |
| 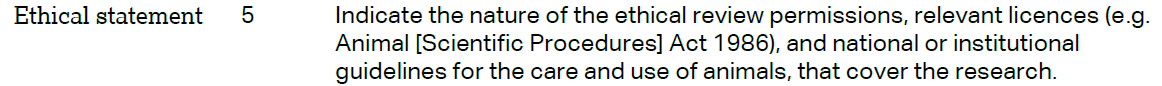 | | | Material & Methods  Paragraph 1 |  |
| 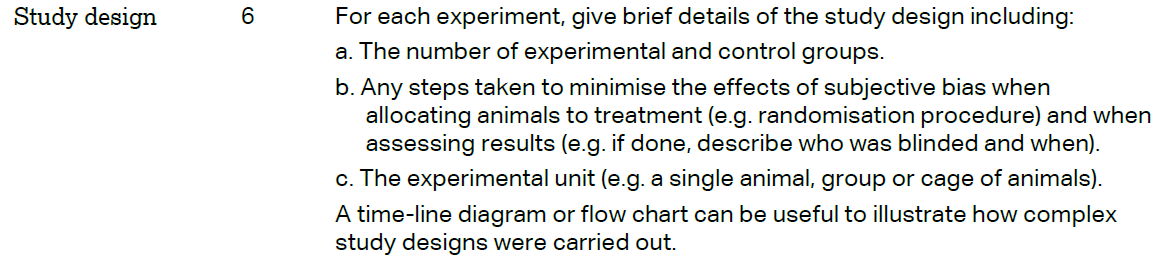 | | | Not applicable.  Only cultured cells were used. |  |
| 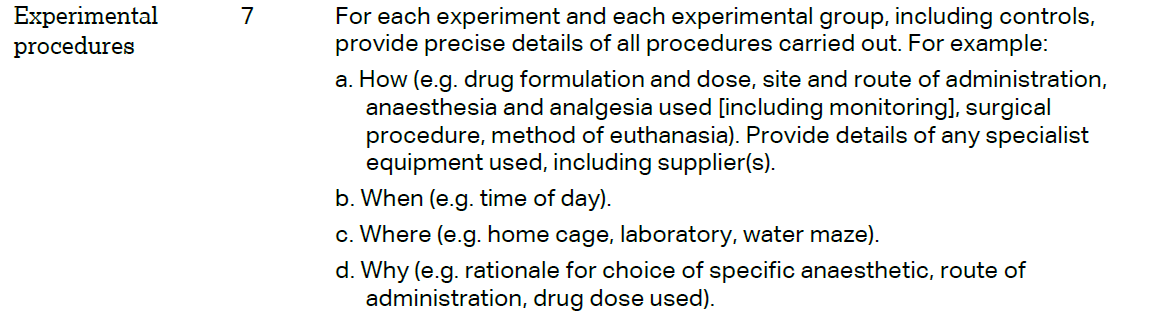 | | | Not applicable.  Only cultured cells were used |  |
| 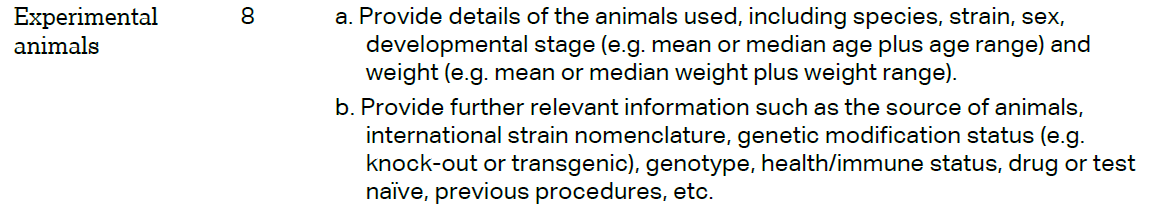 | | | Not applicable.  Only cultured cells were used |  |

The ARRIVE guidelines. Originally published in *PLoS Biology*, June 2010^1^

| 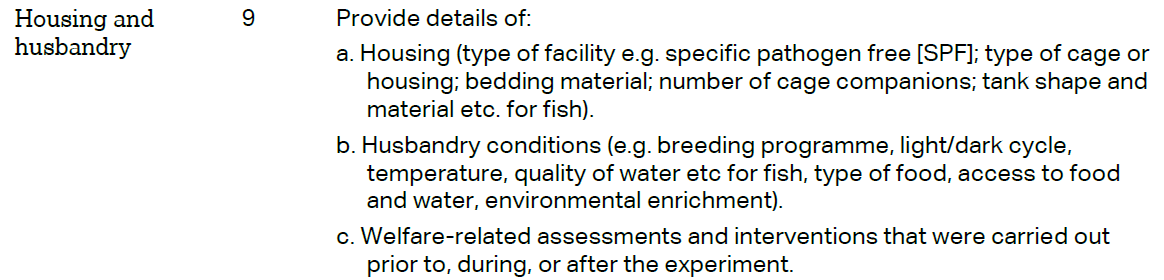 | Not applicable.  Only cultured cells were used | |
| --- | --- | --- |
| 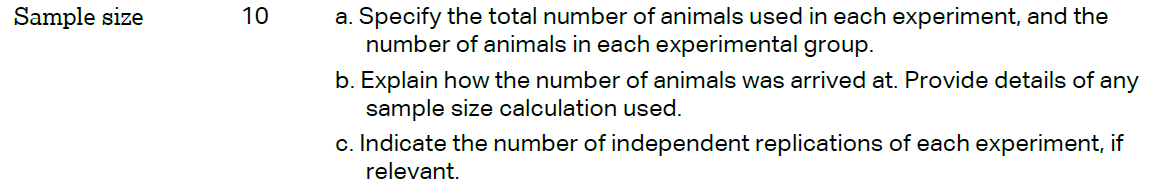 | Not applicable.  Only cultured cells were used | |
| 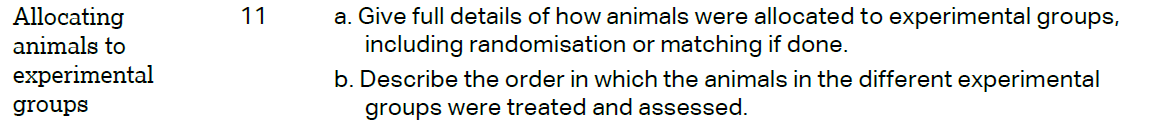 | Not applicable.  Only cultured cells were used | |
| 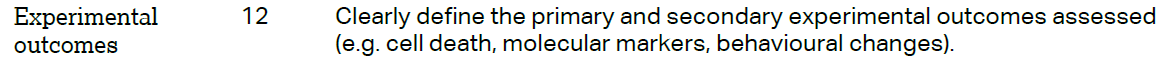 | Cell proliferation | |
| 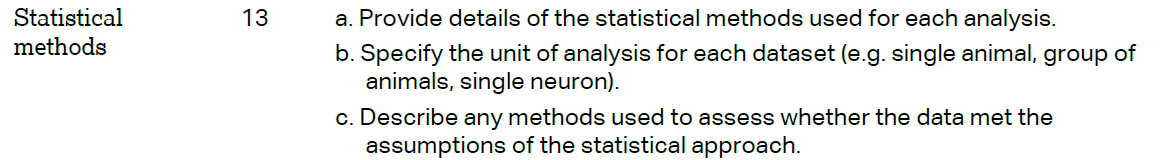 | Material and Methods. In every paragraph and in figure legends. | |
| RESULTS |  | |
| 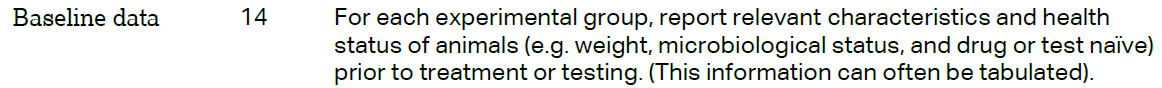 | Not applicable.  Only cultured cells were used | |
| 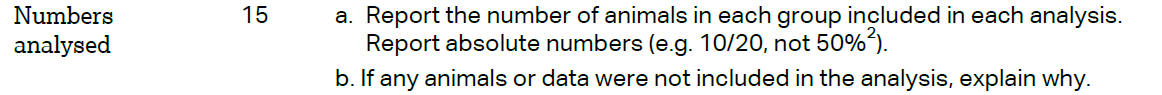 | Not applicable.  Only cultured cells were used | |
| 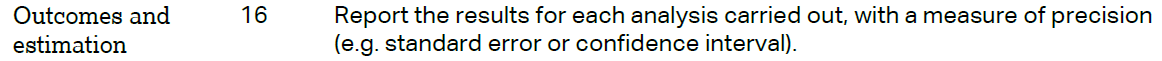 | Results. In every paragraph and in figure legends. | |
| 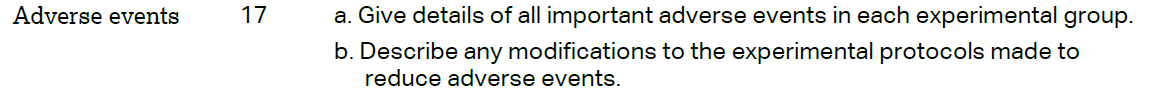 | Not applicable. | |
| DISCUSSION |  | |
| 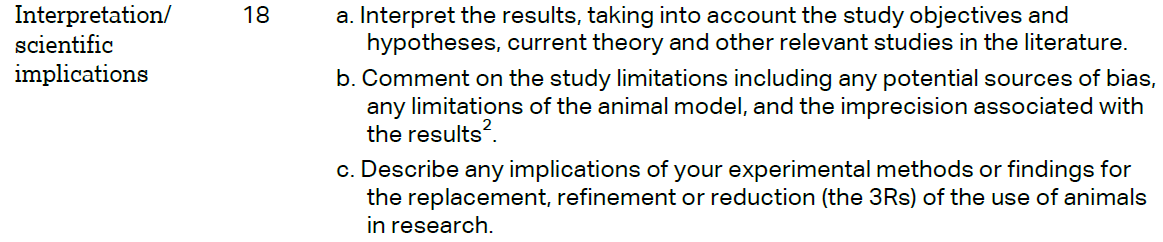 | Disccusion.  Paragraph 1  Not applicable | |
| 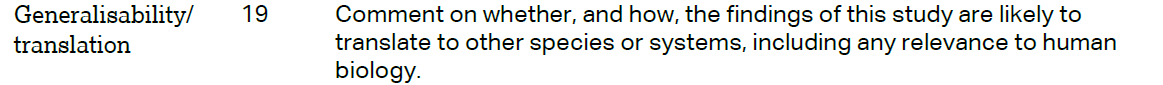 | Not applicable.  Very speculative | |
| 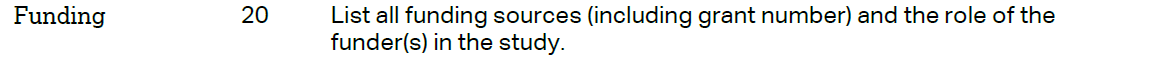 | | Not applicable |


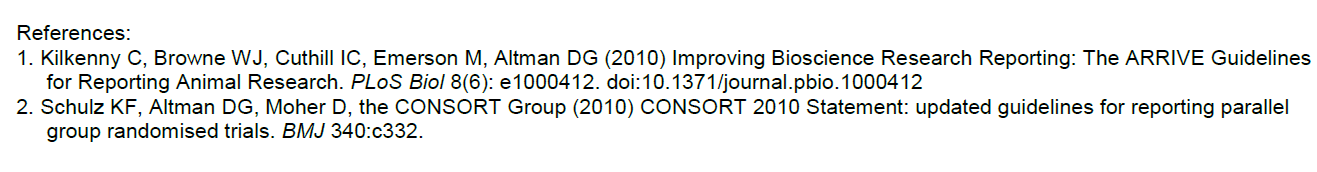

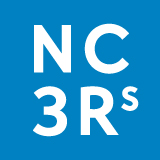

Supplement: S1 Checklist — (DOCX) [file pone.0139619.s001.docx]
